# The risk of classical galactosaemia in newborns with borderline galactose metabolites on newborn screening

**DOI:** 10.1002/jmd2.12339

**Published:** 2022-09-21

**Authors:** Isaac Bernhardt, Emma Glamuzina, Bryony Ryder, Detlef Knoll, Natasha Heather, Mark De Hora, Dianne Webster, Callum Wilson

**Affiliations:** ^1^ National Metabolic Service, Auckland City Hospital and Starship Children's Hospital Auckland New Zealand; ^2^ Chemical Pathology (Section New Born Screening), Auckland City Hospital Auckland New Zealand; ^3^ Newborn Metabolic Screening Unit, Auckland City Hospital Auckland New Zealand

## Abstract

Newborn screening (NBS) for classical galactosaemia (CG) facilitates early diagnosis and treatment to prevent life‐threatening complications, but remains controversial, and screening protocols vary widely between programmes. False‐negatives associated with first‐tier screening of total galactose metabolites (TGAL) are infrequently reported; however, newborns with TGAL below the screening threshold have not been systematically studied. Following the diagnosis of CG in two siblings missed by NBS, a retrospective cohort study of infants with TGAL just below the cut‐off (1.5 mmol/L blood) was conducted. Children born in New Zealand (NZ) from 2011 to 2019, with TGAL 1.0–1.49 mmol/L on NBS were identified from the national metabolic screening programme (NMSP) database, and clinical coding data and medical records were reviewed. *GALT* sequencing was performed if CG could not be excluded following review of medical records. 328 infants with TGAL 1.0–1.49 mmol/L on NBS were identified, of whom 35 had ICD‐10 codes relevant to CG including vomiting, poor feeding, weight loss, failure to thrive, jaundice, hepatitis, *Escherichia coli* urinary tract infection, sepsis, intracranial hypertension and death. CG could be excluded in 34/35, due to documentation of clinical improvement with continued dietary galactose intake, or a clear alternative aetiology. *GALT* sequencing in the remaining individual confirmed Duarte‐variant galactosaemia (DG). In conclusion, undiagnosed CG appears to be rare in those with TGAL 1.0–1.49 mmol/L on NBS; however, our recent experience with missed cases is nevertheless concerning. Further work is required to establish the optimum screening strategy, to maximize the early detection of CG without excess false‐positives.

**Synopsis**

False‐negative newborn screening results for classical galactosaemia are reported in association with primary measurement of total galactose metabolites; however, such patients are likely to present clinically.

## 1 INTRODUCTION

Classical galactosaemia (CG) caused by galactose‐1‐phosphate uridyltransferase (GALT) deficiency occurs secondary to bi‐allelic *GALT* variants (OMIM#230400), at an incidence of ~1/50 000 live‐births in Caucasians.^1^ GALT catalyzes the conversion of galactose‐1‐phosphate to UDP‐galactose in the Leloir pathway, and deficiency leads to accumulation of galactose‐1‐phosphate and galactose among other metabolites.^2^ CG presents in neonates with hepatopathy, cataract, encephalopathy and sepsis, and has high mortality unless recognized and treated with dietary galactose‐restriction.^2^ Early detection by newborn screening (NBS) allows prompt initiation of galactose‐restricted diet, and prevention of life‐threatening disease complications and cataract.^3^, ^4^ However NBS for galactosaemia remains controversial, due to the view that it is readily diagnosed clinically, and that neuro‐developmental complications occur despite treatment.^2^, ^5^, ^6^

Newborn screening approaches for CG vary widely between screening programmes.^7^, ^8^ CG screening protocols are influenced by regional variation in the incidence of CG and non‐classical galactosaemia, service capacity to manage false‐positives, and varied approaches to reporting Duarte‐variant galactosaemia (DG).^7^, ^9^ NBS for galactosaemia was introduced in New Zealand (NZ) in the 1970's. The dried blood spot (DBS) sample is collected at 48–72 h, and is sent to the centralized national metabolic screening programme (NMSP). Total galactose metabolites (TGAL) comprising the sum of galactose and galactose‐1‐phosphate in whole‐blood, is used as the primary marker. Second‐tier analyses are performed if TGAL is above the cut‐off, including measurement of galactose‐1‐phosphate, galactose and GALT activity (quantitative assay).^10^, ^11^ This approach is preferred due to the goal of detecting CG, galactokinase (OMIM#230200) and epimerase (OMIM#230350) deficiency, and to avoid detection of DG.

The NMSP detected 10 patients with CG from 2000 to 2008, and all had significantly elevated TGAL (range 2.24–16.6 mmol/L). The TGAL cut‐off during this period was 0.8 mmol/L, which was associated with a positive predictive value (PPV) well below 10%. Therefore, the cut‐off was increased to 1.5 mmol/L, but remained 0.8 mmol/L for infants in neonatal intensive care, due to potentially inadequate milk intake in this population. In 2019, two siblings with TGAL <1.5 mmol/L were missed by NBS then subsequently diagnosed with CG after presenting clinically with liver failure, raising concern that other CG patients had been missed by the NMSP. A retrospective cohort study of infants with TGAL 1.0–1.49 mmol/L was conducted, to estimate the incidence of false‐negatives associated with a cut‐off of 1.5 mmol/L. It was hypothesized that missed cases would become progressively unwell unless CG was diagnosed clinically, liver‐transplantation was performed, or low‐galactose feeds were started as treatment for an alternative disorder mimicking CG.

## 2 MATERIALS AND METHODS

In NZ all infants are assigned a unique national health identifier (NHI) at birth, which is recorded on the NBS sample and all subsequent healthcare episodes. All NBS results are recorded in the NMSP database. Participation in NBS in NZ is voluntary, with high uptake (>99.5% births). The vast majority of secondary and tertiary healthcare episodes for children in NZ occur at public hospitals, and each healthcare episode is coded according to the International Classification of Disease‐10 (ICD‐10). Therefore significant childhood illnesses requiring hospital care are reliably captured, and ICD‐10 coding data can be readily matched to NBS results as previously described.^12^

TGAL was measured in whole‐blood using a fluorescent galactose oxidase method. NHI numbers were obtained for all NBS samples from July 2011 to December 2019 with TGAL 1.0–1.49 mmol/L. ICD‐10 codes linked to these NHI were obtained from the Ministry of Health (MOH). Coding criteria considered consistent with a missed diagnosis of CG included: galactosaemia, poor feeding, poor growth, weight loss, vomiting, lactose intolerance, neonatal jaundice, liver disease/failure, hepatomegaly, cholestasis, hepatitis, coagulopathy, liver transplantation, sepsis, *Escherichia Coli* infection, cataract, renal tubulopathy, developmental delay, intracranial hypertension, encephalopathy, seizures, coma and death. If relevant coding criteria were present, review of clinical records was conducted to determine if the clinical course was potentially consistent with CG. Documented resolution of symptoms or normal growth and development, in the presence of ongoing dietary galactose intake, was considered to be inconsistent with CG.

If clinical records were potentially consistent with CG, *GALT* sequencing was performed on DNA extracted from the DBS sample. The coding regions of the *GALT* gene, flanking ±20 base‐pairs (encompassing the splice sites) and the 3′ end of the promoter region were sequenced by Sanger‐based sequencing. Analysis of sequence data was performed using Variant Reporter Software v2.

Data were also collected regarding total NBS samples during the study period, and those with TGAL >1.5 mmol/L including true‐positives and false‐positives. A positive screen was defined as an NBS result triggering additional actions including request for repeat DBS samples or clinical referral. The clinical presentation, NBS data and *GALT* sequencing results for known CG patients missed by NBS in the study period are also described.

## 3 RESULTS

**FIGURE 1:**
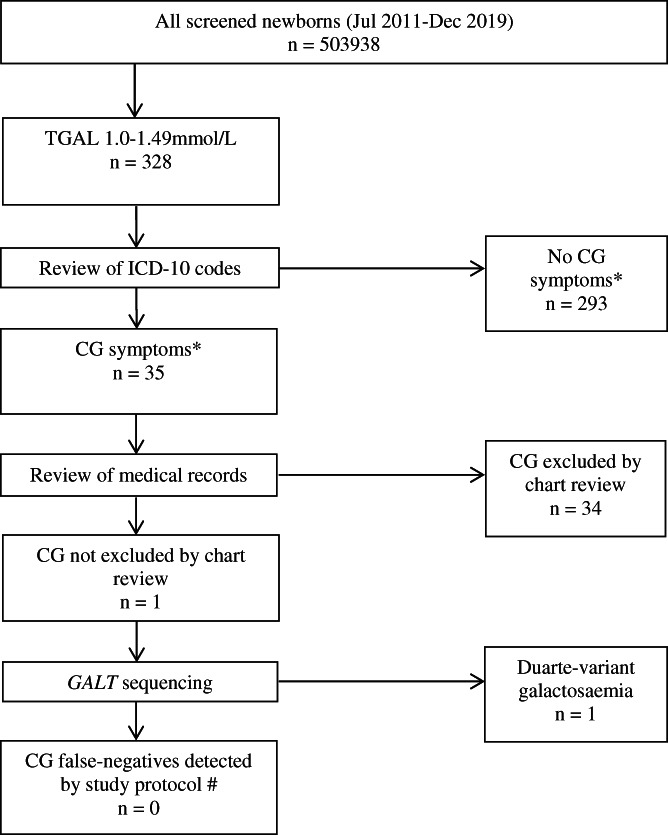
Summary of inclusion and exclusion criteria for cohort selection. CG, classical galactosaemia; ICD‐10, International Classification of Diesease‐10; TGAL, total galactose metabolites. *CG symptoms determined from ICD‐10 codes as described in ‘Methods’. # Excludes clinically‐presenting false‐negative CG cases (Patients A&B)

503 938 infants underwent NBS in the study period. Of these, 27 positive screens for CG were detected. CG was confirmed in only 2/27 (PPV 7.4%). No patients were diagnosed with galactokinase, epimerase or mutarotase deficiency. 328 infants were identified with TGAL 1.0–1.49 mmol/L (Figure 1). Of these, 35 had relevant ICD‐10 codes including: poor feeding (9), neonatal jaundice (5), abnormal weight loss (5), poor weight gain (4), nausea and vomiting (3), sepsis (3), *E. Coli* urinary tract infection (2), coma (2), seizures (1), developmental delay (1), hepatitis (1), intracranial hypertension (1) and death (1). One individual developed intracranial hypertension, and died at 3 years of age, and was found to have a large posterior fossa tumour at post‐mortem. Of the other cases, all but one were noted to have clinical improvement or normal growth and development, with evidence of ongoing regular dietary lactose intake.

One individual was identified for whom CG could not be excluded following review of clinical records. This individual presented to hospital at 6 days of age with 15% weight loss since birth, jaundice, and apnoea. Formula feeds were given by nasogastric tube during this admission. However note was made of a change of formula prior to discharge; unfortunately the new formula preparation was not documented. Subsequent records at 3 years of age documented hearing loss, hypotonia, global developmental delay and normal liver enzymes, but dietary history was not recorded. *GALT* sequencing showed that this individual was heterozygous for the pathogenic c.563A > G, p.(Gln188Arg) variant and the Duarte (D2) variant c.‐119_‐116delGTCA, consistent with DG. This result was not considered relevant to the clinical presentation.

During the study period, two individuals presented clinically with CG, and were missed by NBS. These siblings were born at term, and were breastfed prior to NBS at 2 days of age (Table 1). TGAL was 0.62 mmol/L in the older male sibling (Patient A), and 0.81 mmol/L in the younger female sibling (Patient B).

**TABLE 1:** Summary of the clinical, biochemical and molecular findings in the two cases

	Patient A Missed on NBS and clinically as repeat testing was taken on intravenous fluids and after red cell transfusion	Patient B (Sibling of Patient A who was 13 months at the time of her birth, well and had no diagnosis)
Sex	Male	Female
Gestational age at birth	41 weeks	38 weeks + 6 days
Birthweight	4.2 kg	3.6 kg
Feeds prior to NBS	Breastmilk	Breastmilk
NBS TGAL (2 days of age; cut off 1.5 mmol/L)	0.62 mmol/L	0.81 mmol/L
Feeds prior to presentation	Standard cow's milk infant formula	Breastmilk
Age at presentation	4 weeks	10 days
Presenting symptoms	Poor growth, liver failure with jaundice and severe ascites	Weight loss, liver failure with coagulopathy and hyperammonaemia
TGAL at presentation	1.28 mmol/L^a^	9.2 mmol/L^b^
Galactose (normal < 1.35 mmol/L)	0.9 mmol/L^a^	9.2 mmol/L^b^
Gal‐1‐P (normal < 1.35 mmol/L)	0.62 mmol/L^a^	0.0 mmol/L^b^
GALT activity (quantitative assay)^8^, ^9^	43%^a^	6%^b^
Initial treatment	Extensively hydrolysed formula (high MCT, low galactose) for unexplained conjugated jaundice	Extensively hydrolysed formula (high MCT, low galactose)
Progress following initial treatment	All presenting symptoms resolved	Coagulation and ammonia normalized, growth and liver enzymes improved
Age at diagnosis	14 months (diagnosed following diagnosis of younger sibling, Patient B)	6 weeks
*GALT* genotype	Homozygous c.563A > G, p.Gln188Arg	Homozygous c.563A > G, p.Gln188Arg
Treatment following CG diagnosis	Galactose‐restricted diet including soy formula	Galactose‐restricted diet including soy formula
Repeat GALT activity at diagnosis^c^	<5%	<5%
Gal‐1‐P on low‐galactose feeds following diagnosis	0.17 mmol/L	0.24 mmol/L
Outcome	4 years of age: liver disease resolved, mild bilateral cataracts, subtle issues with behavior and speech development	3 years of age; liver disease resolved, normal growth and development

^a^ At 35 days of age, on intravenous fluids for 4 days prior, and post‐transfusion with red cells.

^b^ At 17 days of age while receiving breastfeeds, no preceding transfusion.

^c^ Repeat GALT assay using alternative method: GSP kit from PerkinElmer. [Correction added on 08 October 2022 after first online publication: Table 1 has been updated in this version.]

Patient A was fed with standard infant formula containing lactose prior to presentation with liver failure at 4 weeks of age. TGAL was 1.28 mmol/L at 35 days of age, with galactose 0.9 mmol/L, galactose‐1‐phosphate 0.62 mmol/L and GALT activity 43% of the mean normal value. He had received intravenous fluids and red cell transfusion in the preceding 4 days, thus explaining the normal GALT activity. Extensively hydrolysed formula feeds were started for management of cholestasis, due to their high medium‐chain triglyceride (MCT) content; however, this preparation was also low in galactose. He made a full clinical recovery with no evidence of liver disease or cataract at 14 months of age.

Patient B was born 13 months after Patient A, and presented with liver failure at 10 days of age. TGAL was 9.2 mmol/L at 17 days with GALT activity <10% of the mean normal value. Whole exome sequencing in Patient B identified the homozygous *GALT* variant c.563A > G, p.(Gln188Arg), which was then detected in Patient A by Sanger sequencing.

## 4 DISCUSSION

Population NBS for CG has been feasible since 1964,^6^ and is now undertaken by many screening programmes. Screening protocols vary significantly between centres, and include primary measurement of either GALT activity or galactose metabolites (TGAL), with variable screening thresholds.^7^, ^8^, ^13^ First‐tier TGAL measurement enables detection of galactokinase, epimerase and the recently described mutarotase deficiency (OMIM#618881), in addition to CG.^14^, ^15^ However TGAL may also be raised in DG, heterozygous CG carriers, neonatal liver disease and other rare inherited metabolic disorders, and therefore second‐tier analysis of galactose metabolites and GALT activity is used to increase specificity.^15^ The laboratory burden of second‐tier testing is subject to the TGAL cut‐off used; however, false‐positives occur at a significant rate despite second‐tier testing.^4^ False‐negatives associated with primary TGAL screening are thought to be rare, but have been reported in association with hypomorphic *GALT* alleles, or when galactose intake prior to sampling is significantly limited.^8^, ^16^, ^17^ To our knowledge, this is the first systematic study of infants with TGAL below the screening threshold.

In this cohort of 328 newborns with TGAL in the range of 1.0–1.49 mmol/L, no missed cases of CG were identified, and therefore the risk of undiagnosed CG in this group appears to be low. It is unlikely but plausible that further individuals with CG in this cohort escaped detection by this review; for example, if low‐galactose feeds were initiated in primary care for another indication, in a newborn with very mild symptoms not requiring hospital care.

The primary limitation of this study is that *GALT* sequencing was only performed in one individual, who was found to have DG. It is likely that many others also had DG, or were heterozygous CG carriers. However neither DG or CG‐heterozygosity is associated with short‐ or long‐term medical complications.^9^, ^18^, ^19^, ^20^ Therefore their detection by NBS is undesirable, and genetic testing for these entities was not considered to be clinically relevant or ethically justifiable. While no individuals with cataract were identified in this cohort, testing for variants in *GALK1* or *GALM* may also have been informative; however, detection of galactokinase or mutarotase deficiency was not the purpose of this study.

Newborns with TGAL just below the screening threshold of 1.5 mmol/L were selected for inclusion, due to the presumed higher risk of false‐negatives in this group. However it is noteworthy that the two index cases missed on NBS had TGAL well below the cut‐off and were not captured by the study criteria. It is also noteworthy that while both siblings were homozygous for the common pathogenic c.563A > G, p.(Gln188Arg) variant associated with severe disease,^5^ Patient A had an unusually late presentation despite ongoing dietary galactose intake. While no known risk factors for false‐negative screening were detected in these patients, it is peculiar that they were siblings, and it is therefore possible that other shared genetic or environmental factors contributed to an attenuated elevation of TGAL at the time of NBS. Unfortunately, diagnostic delay in Patient A was further compounded by falsely normal repeat biochemistry, due to preceding fluid and transfusion therapy.

The prevalence of CG diagnosed on NBS in the study period was unexpectedly low. 19 individuals were diagnosed with CG in NZ over 20 years (incidence 1/63 000 live‐births), consistent with the expected population incidence.^1^ However during the study period the observed incidence was 4/503 938 (1/125 000), and 2/4 were missed by NBS (sensitivity 50%). While no additional missed cases were identified in this study, the low detection rate of CG by NBS over this period is cause for concern. A survey of other Australasian screening programmes, of which 4/5 undertake NBS for CG, revealed five additional missed cases (TGAL range 0.71–1.09 mmol/L). Of these patients, only 2/5 were not receiving galactose feeds prior to NBS sample collection at 2–3 days of age. These programmes have all lowered their cut‐offs, with three now using 0.5 mmol/L and one using 1.0 mmol/L. While a screening threshold of 0.5 mmol/L would detect the index cases presented here, it would significantly increase second‐tier testing and false‐positives (including DG). Reporting of false positive NBS results may cause significant distress for the family of an affected infant, and this recognized harm of NBS needs to be balanced against the benefits of increased detection of CG.^21^

Beyond Australasia, the GALT assay is more commonly used as the primary screening test, and false‐negatives using this approach appear to be rare.^6^, ^7^, ^13^ Although false‐negative GALT assays are known to occur following neonatal transfusion,^6^ this is likely to be an increasingly uncommon scenario.^22^ Use of a primary GALT approach is associated with a significant burden of false‐positives, especially during summer months due to heat degradation of the DBS sample.^23^ Additionally, it does not detect non‐classical galactosaemia due to galactokinase, epimerase or mutarotase deficiency, which are secondary targets of NBS using a primary TGAL approach. Both approaches merit consideration, and the NMSP has implemented an interim TGAL cut‐off of 0.8 mmol/L.

Finally, the authors acknowledge that large‐scale prospective studies confirming the benefit of NBS for CG are lacking.^8^, ^24^ However early treatment is associated with reduced mortality in uncontrolled studies.^3^, ^25^ In the experience of the NMSP, NBS facilitates timely detection and treatment, particularly with regards to early identification of coagulopathy, which if unrecognized, has resulted in fatal outcome.

## 5 CONCLUSION

Clinically presenting patients with CG, with TGAL well below cut‐off, have been missed by the NMSP and other screening programmes that utilize a primary TGAL method. In this retrospective cohort study of newborns with TGAL 1.0–1.49 mmol/L on NBS, no additional missed cases of CG were identified. This suggests that false‐negatives associated with TGAL in this range are not common, and such patients are likely to present clinically. The occurrence of clinically presenting false‐negative cases with TGAL between 0.5–1.0 mmol/L is nevertheless concerning, and warrants consideration of a significantly lower threshold or alternative screening strategy. NBS for CG may significantly reduce morbidity and mortality in affected infants; however, the optimum screening strategy and thresholds remain to be clarified.

## CONFLICT OF INTEREST

Isaac Bernhardt declares no conflict of interest. Emma Glamuzina declares no conflict of interest. Bryony Ryder declares no conflict of interest. Detlef Knoll declares no conflict of interest. Natasha Heather declares no conflict of interest. Mark De Hora declares no conflict of interest. Dianne Webster declares no conflict of interest. Callum Wilson declares no conflict of interest.

## ETHICAL STATEMENT/INFORMED CONSENT

Participation for Newborn Screening (NBS) in New Zealand is voluntary. Since July 2011, routine informed consent for NBS has included consent for additional uses of the dried blood‐spot sample, for activities including quality control and audit by the National Metabolic Screening Programme, as well as for research approved by an ethics committee and the Ministry of Health. Therefore, only samples obtained subsequent to the implementation of this policy were included. This study was approved by the Health and Disability Ethics Committee. All procedures followed were in accordance with the ethical standards of the responsible committee on human experimentation (institutional and national) and with the Helsinki Declaration of 1975, as revised in 2000 (5). Written consent for publication of de‐identified information has been obtained from the parent/guardian of Case A and Case B.

## Data Availability

Data archiving is not mandated but data will be made available on reasonable request.
